# Associations between hypertension and the peroxisome proliferator-activated receptor-δ (*PPARD*) gene rs7770619 C>T polymorphism in a Korean population

**DOI:** 10.1186/s40246-018-0162-6

**Published:** 2018-06-18

**Authors:** Minjoo Kim, Minkyung Kim, Hye Jin Yoo, Jayoung Shon, Jong Ho Lee

**Affiliations:** 1 Research Center for Silver Science, Institute of Symbiotic Life-TECH, Yonsei University, Seoul, 03722 Korea; 2 Department of Food and Nutrition, Brain Korea 21 PLUS Project, College of Human Ecology, Yonsei University, 50 Yonsei-ro, Seodaemun-gu, Seoul, 03722 Korea; 3 Department of Food and Nutrition, National Leading Research Laboratory of Clinical Nutrigenetics/Nutrigenomics, College of Human Ecology, Yonsei University, Seoul, 03722 Korea

**Keywords:** Peroxisome proliferator-activated receptor-δ gene, Genetic polymorphism, Blood pressure, Hypertension, Malondialdehyde

## Abstract

**Background:**

Oxidative stress is associated with the increased risk of hypertension (HTN). This cross-sectional study is aimed to identify the association between the peroxisome proliferator-activated receptor-δ (*PPARD*) polymorphism and plasma malondialdehyde (MDA), an oxidative stress marker which is related to HTN development, and to determine whether *PPARD* gene is a candidate gene for HTN.

**Results:**

One thousand seven hundred ninety-three individuals with normal blood pressure (BP) and HTN were included in this cross-sectional study. The Korean Chip was used to obtain genotype data. Through the analysis, the ten most strongly associated single-nucleotide polymorphisms (SNPs) were nominated for an MDA-related SNP. Among them, the rs7770619 polymorphism was identified in the *PPARD* gene. The CT genotype of the *PPARD* rs7770619 C>T polymorphism was associated with a lower risk of HTN before and after adjustments for age, sex, body mass index, smoking, and drinking. Significant associations were observed between plasma MDA and the *PPARD* rs7770619 C>T polymorphism and between systolic BP and the *PPARD* rs7770619 SNP in the controls. The CT controls showed significantly lower systolic BP and plasma MDA than the CC controls. Additionally, in both controls and HTN patients, the CT subjects showed significantly lower serum glucose and higher adiponectin levels than the CC subjects. Furthermore, the CT subjects showed significantly higher serum free fatty acid levels than the CC subjects among the HTN patients.

**Conclusion:**

This is a new finding that the *PPARD* rs7770619 C>T SNP is a novel candidate variant for HTN based on the association between *PPARD* and plasma MDA in a Korean population.

**Electronic supplementary material:**

The online version of this article (10.1186/s40246-018-0162-6) contains supplementary material, which is available to authorized users.

## Background

Oxidative stress is defined as a sustained increase in the levels of reactive oxygen species (ROS), such as hydrogen peroxide, superoxide anion radicals, and other free radicals. Lipids have been reported as one of the primary targets of ROS. Lipid peroxidation produces highly reactive aldehydes, including malondialdehyde (MDA), which has been reported as a primary biomarker of free radical-mediated lipid damage and oxidative stress [1]. Increased MDA levels as a marker of oxidative stress were higher in hypertensive patients than in normotensive individuals [2, 3]. Additionally, a positive correlation between serum MDA levels and systolic and diastolic blood pressure (BP) has been reported [4].

Hypertension (HTN) is a multifactorial disorder involving both genetic and environmental factors [5]. Therefore, genetic factors affecting oxidative stress may include a common genetic basis of susceptibility to HTN. Although some studies have focused on the association between peroxisome proliferator-activated receptor (PPAR) and HTN [6–8], the association between PPAR-δ (PPARD) and HTN has not been extensively studied previously. Indeed, PPARD has been suggested to regulate BP by modulating risk factors of HTN, including obesity and fatty acid catabolism [9]. Since a close relationship was observed between BP and MDA levels in HTN [2, 3], the MDA-related single-nucleotide polymorphisms (SNPs) analyzed with the Korean Chip (K-CHIP) could also be novel SNPs associated with HTN risk. The K-CHIP is a customized chip optimized for genetic studies on diseases and complex traits in the Korean population. Therefore, the objective of this study was to determine whether the *PPARD* gene is a candidate gene for HTN by identifying any association between *PPARD* and MDA, which is increased in HTN [2, 3].

## Methods

### Study population

All individuals who visited the Health Service Center (HSC) at National Health Insurance Corporation Ilsan Hospital, Goyang, Korea, for their routine checkups (from January 2010 to March 2015) were potential study subjects for this research. Based on the data screened from HSC, men and women aged over 20 years (adult subjects) with nondiabetic normotension (systolic BP < 140 mmHg and diastolic BP < 90 mmHg) or HTN (systolic BP ≥ 140 mmHg or diastolic BP ≥ 90 mmHg) were asked to participate in this study and were given detailed explanation regarding the study, and then, individuals who agreed to take part in the study were recruited. These potential subjects were referred to the Department of Family Medicine, and their health and BP were reexamined. Finally, individuals who met the study criteria were included (*n* = 2167). The exclusion criteria were a current diagnosis or history of cardiovascular disease, liver disease, renal disease, pancreatitis, or cancer; pregnancy or lactation; and regular use of any medication except for HTN treatments. The inclusion criteria were men and women adults (aged over 20 years), nondiabetic (fasting glucose < 126 mg/dL and no use of glucose-lowering medication), and individuals who do not correspond to the exclusion criteria (Additional file 1: Figure S1). The aim of the study was carefully explained to all participants, who provided their written informed consent. The Institutional Review Board of Yonsei University and the National Health Insurance Corporation Ilsan Hospital approved the study protocol, which complied with the Declaration of Helsinki.

### Blood sample collection

Venous blood samples were collected following an overnight fast for at least 12 h. The fasting blood specimens were collected in EDTA-treated tubes and serum tubes (BD Vacutainer; Becton, Dickinson and Company, Franklin Lakes, NJ, USA) and were then centrifuged (1200 rpm, 20 min, 4 °C) to obtain plasma and serum. The plasma and serum sample aliquots were stored at − 80 °C prior to analysis.

### BP measurement

Systolic and diastolic BP were measured using a random-zero sphygmomanometer (HM-1101, Hico Medical Co., Ltd., Chiba, Japan) with appropriately sized cuffs after a rest period of at least 20 min in a seated position. BP was measured three times in both arms. The differences among the three systolic BP measurements were always less than 2 mmHg. Participants were instructed not to smoke or drink alcohol for at least 30 min before each BP measurement.

### Clinical and biochemical assessments

Body weight (UM0703581; Tanita, Tokyo, Japan) and height (GL-150; G-tech International, Uijeongbu, Korea) were measured after subjects removed their shoes, and the body mass index (BMI) was calculated (kg/m^2^).

The serum fasting triglyceride (TG) and total cholesterol (TC) levels were measured enzymatically using TG and CHOL Kits (Roche, Mannheim, Germany), respectively. Serum fasting high-density lipoprotein (HDL)-cholesterol was measured by a selective inhibition method with an HDL-C Plus Kit (Roche, Mannheim, Germany). The resulting color reactions of the assays were monitored using a Hitachi 7600 autoanalyzer (Hitachi, Tokyo, Japan). Low-density lipoprotein (LDL)-cholesterol values were obtained indirectly using the Friedewald formula: LDL-cholesterol = TC − [HDL-cholesterol + (TG/5)]. Serum fasting free fatty acid was measured with enzymatic assays using an NEFA-M Kit (Shinyang Diagnostics, Gyeonggi, Korea), and the resulting color reactions of the assays were monitored with a Hitachi 7600 autoanalyzer (Hitachi, Tokyo, Japan).

The serum fasting glucose level was measured by a hexokinase method using a GLU Kit (Roche, Mannheim, Germany). The serum fasting insulin was measured by an immunoradiometric assay using an Insulin IRMA Kit (DIAsource, Louvain, Belgium). The resulting color reaction was monitored with a Hitachi 7600 autoanalyzer (Hitachi, Tokyo, Japan) and an SR-300 system (Stratec, Birkenfeld, Germany), respectively. To calculate insulin resistance (IR), the equation for homeostatic model assessment (HOMA) was used: HOMA-IR = [fasting insulin (μIU/mL) × fasting glucose (mg/dL)]/405. Plasma adiponectin was measured via an enzyme immunoassay using a Human Adiponectin ELISA Kit (B-Bridge International Inc., San Jose, CA, USA), and the resulting color reaction was monitored with a Victor2 (PerkinElmer Life Sciences, Turku, Finland).

Serum high-sensitivity C-reactive protein (hs-CRP) levels were measured using a CRP Kit (Roche, Mannheim, Germany), and the resulting colorimetric reaction was monitored with a Hitachi 7600 autoanalyzer (Hitachi, Tokyo, Japan). Plasma MDA was measured from thiobarbituric acid reactive substances (TBARS) using a TBARS Assay Kit (ZeptoMetrix Co., Buffalo, NY, USA).

### Affymetrix Axiom™ KORV1.0-96 Array hybridization and SNP selection

The detailed information for this protocol is described in our previous study [10]. A total of 2167 samples were genotyped according to the manufacturer’s protocol, which recommended the Axiom® 2.0 Reagent Kit (Affymetrix Axiom® 2.0 Assay User Guide; Affymetrix, Santa Clara, CA, USA). The genotype data were produced using the K-CHIP, which was available through the K-CHIP consortium. The K-CHIP was designed by the Center for Genome Science at the Korea National Institute of Health (4845-301, 3000-3031).

Samples that revealed the following features were excluded during the quality control process: sex inconsistency, markers with a high missing rate (> 5%), individuals with a high missing rate (> 10%), minor allele frequency < 0.01, and a significant deviation from Hardy-Weinberg equilibrium (HWE) (*p* < 0.001). In addition, SNPs were excluded if they were related to each other in linkage disequilibrium. Consequently, among a total of 833,535 SNPs on the arrays and 2167 samples, 395,787 SNPs and 2158 samples remained, and they were used in subsequent association analyses.

### Statistical analysis

HWE and association assessments between SNPs and MDA using linear regression analysis were performed in PLINK version 1.07 (http://zzz.bwh.harvard.edu/plink); for issues of multiple comparisons between SNPs and MDA, false discovery rate (FDR) correction was used. Descriptive statistical analyses were conducted using SPSS version 23.0 (IBM, Chicago, IL, USA). Logarithmic transformation was used for the skewed variables, and a two-tailed *p* value of < 0.05 was considered statistically significant. An independent *t* test was performed on the continuous variables. Sex distribution, smoking and drinking status, and genotype frequency were tested using the chi-squared test. The association of HTN with a genotype was calculated using the odds ratio (OR) [95% confidence interval (CI)] of a logistic regression model with an adjustment for confounding factors.

## Results

Through the subsequent analysis using 395787 SNPs and 2158 samples, the ten SNPs that were most strongly associated with plasma MDA were nominated (Additional file 1: Table S1). Among them, one SNP, rs7770619, was identified in the *PPARD* gene. Therefore, we conducted an association analysis of *PPARD* rs7770619 polymorphism. Among 2158 subjects, 313 and 52 subjects did not have data of plasma MDA and rs7770619, respectively; thus, a total of 1793 subjects who had both plasma MDA and rs7770619 data were finally included in the final analysis (Additional file 1: Figure S1).

The clinical and biochemical characteristics of the normotensive controls (*n* = 1359) and HTN patients (*n* = 434) are shown in Table 1. HTN patients included those who use antihypertensive medication (35.9%). Thus, we subdivided HTN patients into two groups: those not treated with antihypertensive drugs (HTN without treatment, *n* = 278) and those treated with antihypertensive drugs (HTN with treatment, *n* = 156). Compared with normotensive controls, both HTN subgroups were older and heavier. After adjusting for age, sex, BMI, smoking, and drinking, the patients in both HTN subgroups showed higher systolic and diastolic BP and plasma MDA than normotensive controls. Serum TG was higher in HTN patients without treatment than in normotensive controls. The HTN with treatment subgroup showed lower TC and LDL-cholesterol and higher glucose than normotensive controls (Table 1).

**Table 1:** Clinical and biochemical characteristics in normotensive controls and HTN patients

	Normotensive controls (*n* = 1359)	HTN patients (*n* = 434)		
		Total (*n* = 434)	HTN without treatment (*n* = 278)	HTN with treatment (*n* = 156)
Age (year)	48.4 ± 0.28	53.8 ± 0.52^***^	51.5 ± 0.66^***^	57.9 ± 0.73^***^
Male/female, *n* (%)	499 (36.7)/860 (63.3)	229 (52.8)/205 (47.2)^***^	153 (55.0)/125 (45.0)^***^	76 (48.7)/80 (51.3)^**^
Current smoker, *n* (%)	193 (14.2)	74 (17.1)	55 (19.8)^*^	19 (12.2)
Current drinker, *n* (%)	824 (60.6)	259 (59.7)	177 (63.7)	82 (52.6)
BMI (kg/m^2^)	23.8 ± 0.08	25.4 ± 0.15^***^	25.3 ± 0.19^***^	25.5 ± 0.23^***^
Systolic BP (mmHg)	116.5 ± 0.31	139.2 ± 0.75^*†††*^	144.4 ± 0.75^*†††*^	129.9 ± 1.31^*†††*^
Diastolic BP (mmHg)	72.9 ± 0.23	88.3 ± 0.49^*†††*^	92.6 ± 0.46^*†††*^	80.6 ± 0.77^*†††*^
Triglyceride (mg/dL)^*∮*^	119.2 ± 1.88	148.3 ± 4.24^*†*^	152.3 ± 5.62^*††*^	141.2 ± 6.20
Total cholesterol (mg/dL)^*∮*^	199.4 ± 0.98	199.3 ± 1.73^*†*^	202.9 ± 2.14	192.8 ± 2.86^*†††*^
HDL-cholesterol (mg/dL)^*∮*^	54.4 ± 0.37	51.2 ± 0.60	51.1 ± 0.74	51.5 ± 1.05
LDL-cholesterol (mg/dL)^*∮*^	122.0 ± 0.90	119.2 ± 1.58^*†††*^	122.3 ± 1.98	113.7 ± 2.55^*†††*^
Glucose (mg/dL)^*∮*^	95.4 ± 0.55	102.5 ± 1.10^*†*^	100.9 ± 1.39	105.3 ± 1.80^*††*^
Insulin (μIU/mL)^*∮*^	8.92 ± 0.12	9.59 ± 0.28	9.83 ± 0.38	9.11 ± 0.36
Free fatty acids (μEq/L)^*∮*^	552.8 ± 6.65	567.9 ± 12.6	560.9 ± 16.0	581.8 ± 20.4
HOMA-IR^*∮*^	2.09 ± 0.03	2.46 ± 0.10	2.54 ± 0.14	2.30 ± 0.10
hs-CRP (mg/dL)^*∮*^	1.28 ± 0.08	1.60 ± 0.15	1.59 ± 0.16	1.61 ± 0.28
Adiponectin (ng/mL)^*∮*^	6.49 ± 0.10	5.98 ± 0.17	5.94 ± 0.22	6.06 ± 0.27
Malondialdehyde (nmol/mL)^*∮*^	8.89 ± 0.09	9.78 ± 0.27	9.33 ± 0.35^*††*^	10.6 ± 0.43^*††*^

Mean ± SE. ^*∮*^Tested following logarithmic transformation. ^*^p < 0.05, ^**^p < 0.01, and ^***^p < 0.001 derived from an independent *t* test between normotensive controls and each subgroup of hypertensive patients. ^*†*^*p* < 0.05, ^*††*^*p* < 0.01, and ^*†††*^*p* < 0.001 derived after adjusting for age, sex, BMI, smoking, and drinking

### Distribution of the *PPARD* rs7770619 C>T polymorphism

The observed and expected frequencies of the *PPARD* rs7770619 C>T polymorphism were in HWE in the entire population and in the control and patient groups. The relative *PPARD* rs7770619 C>T genotypes in the HTN patients differed significantly from those in the normotensive controls (Table 2). There was no homozygous mutation TT genotype in either the normotensive controls or the HTN patients. Frequencies of the T allele of the *PPARD* rs7770619 C>T polymorphism in the HTN patients (0.012) were significantly lower than those in the normotensive controls (0.028) (*p =* 0.007) (Table 2).

**Table 2:** Frequencies of the *PPARD* rs7770619 genotypes in the normotensive controls and the HTN patients

	Normotensive controls (*n* = 1359)		HTN patients (*n* = 434)		*p* values
*PPARD* rs7770619	*n*	%	*n*	%	
CC	1284	94.5	424	97.7	0.006
CT	75	5.5	10	2.3	
T allele frequency	75	2.8	10	1.2	0.007

A chi-squared test was used to calculate the *p* values

The presence of the CT genotype of the *PPARD* rs7770619 C>T SNP was associated with a lower risk of HTN [OR 0.404 (95% CI 0.207–0.788), *p* = 0.008] (Table 3). The significance of the association remained after adjusting for age, sex, BMI, smoking, and drinking [OR 0.478 (95% CI 0.238–0.960), *p* = 0.038], and the *p* value of Hosmer-Lemeshow goodness-of-fit test was 0.700 for this model implying that our model is well-fitted.

**Table 3:** Unadjusted and adjusted odds ratios (ORs) for all the HTN patients according to the *PPARD* rs7770619 genotypes

	HTN patients (*n* = 434)	*p* values
*PPARD* rs7770619	OR (95% CI)	
Model 1		
C^*‡*^ compared with T	0.411 (0.211 to 0.798)	0.009
CC^*‡*^ compared with CT	0.404 (0.207 to 0.788)	0.008
Model 2		
C^*‡*^ compared with T	0.486 (0.243 to 0.970)	0.041
CC^*‡*^ compared with CT	0.478 (0.238 to 0.960)	0.038

Model 1: unadjusted; Model 2: adjusted for age, sex, BMI, smoking, and drinking. *CI* confidence interval. ^*‡*^Reference

### Associations of plasma MDA, systolic BP, serum glucose, free fatty acids, and adiponectin with the *PPARD* rs7770619 C>T polymorphism

**Fig. 1:**
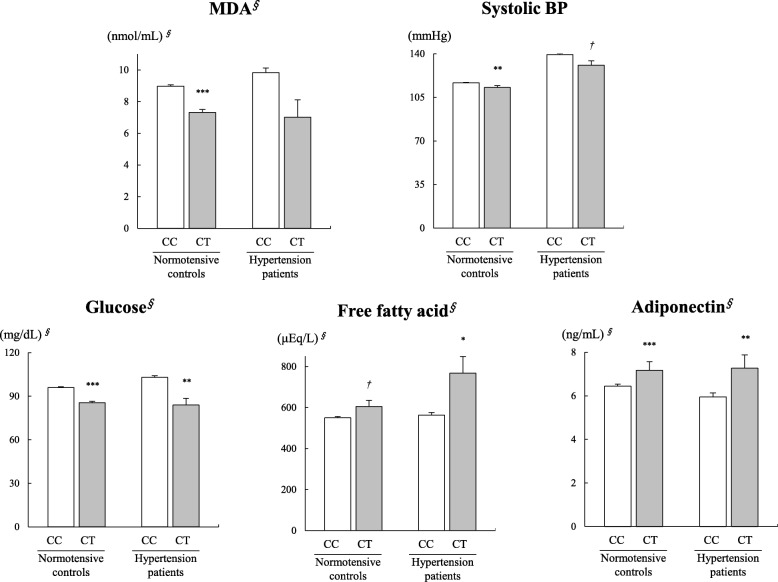
Associations of MDA, systolic BP, glucose, free fatty acid, and adiponectin with the *PPARD* rs7770619 C>T polymorphism. Mean ± SE. The contour integral symbol indicates those tested following logarithmic transformation. ^*†*^*p* < 0.1, ^*^p < 0.05, ^**^p < 0.01, and ^***^p < 0.001 derived from an independent *t* test within each group

A significant association was observed between plasma MDA and the *PPARD* rs7770619 C>T polymorphism in the normotensive controls. The CT carriers showed significantly lower MDA than the CC carriers (CC 8.98 ± 0.09 nmol/mL, CT 7.31 ± 0.20 nmol/mL; *p* < 0.001) (Fig. 1). Similarly, in the HTN patients, the CT carriers showed lower MDA than the CC carriers, but the difference was not statistically significant. Systolic BP and the *PPARD* rs7770619 C>T polymorphism were significantly associated in the normotensive controls (CC 116.7 ± 0.32 mmHg, CT 113.0 ± 1.36 mmHg; *p =* 0.007), and there was a trend toward an association between systolic BP and the *PPARD* rs7770619 C>T polymorphism in the HTN patients (CC 139.4 ± 0.76 mmHg, CT 130.9 ± 3.43 mmHg; *p =* 0.090). In the normotensive controls, the CT carriers showed significantly lower systolic BP than the CC carriers (Fig. 1). Additionally, in both normotensive controls and HTN patients, the CT carriers showed lower glucose and higher adiponectin than the CC carriers. In the HTN patients, compared with the CC carriers, the CT carriers showed higher free fatty acid. In the normotensive controls, there was a trend toward an increase of free fatty acids in the CT carriers compared with the CC carriers (Fig. 1).

## Discussion

The major finding of the present study was that the frequency of the *PPARD* rs7770619 CT genotype was significantly lower in patients with HTN than in the normotensive controls, suggesting that there was an association between the *PPARD* rs7770619 C>T SNP and HTN. This observation correlated with recent findings that the *PPARD* polymorphism has a key role for HTN development [9]. The significance of the present observations was established by the identification of the human polymorphism in the *PPARD* locus with altered BP and plasma MDA levels, which are reliable oxidative stress markers in HTN [2, 3].

Patients with HTN tend to have several conditions that accelerate the atherogenic process, including an increase in free radicals. ROS are the most important free radicals in the human body and cause increased oxidative stress and tissue injury under pathological conditions [11, 12]. Several studies have reported evidence for enhanced ROS production and decreases in the antioxidant reserves in the plasma and tissues of hypertensive animals and humans [13, 14]. MDA is produced during the attack of ROS upon membrane lipoproteins and polyunsaturated fatty acids. Kashyap et al. [15] have reported increased MDA levels in hypertensive subjects compared with those in normotensive subjects and suggested that elevated lipid peroxidation reflected increased oxidative stress in patients with HTN. In a recent study, similar results were obtained in terms of MDA [3]. The authors found significantly higher MDA levels in the essential HTN group than in the control group. Similarly, this study also showed higher MDA levels in the HTN group regardless of whether they were taking antihypertensive medication than in the control group.

In the present study, subjects with the *PPARD* rs7770619 CT genotype showed significantly lower systolic BP than those with the CC genotype in the normotensive controls. Additionally, the significantly lower concentrations of plasma MDA in the subjects carrying the *PPARD* rs7770619 CT genotype than in the subjects with the CC genotype in the normotensive controls and the tendency toward a decrease in the HTN patients were found in this study. Recently, Li et al. [9] have observed associations between rs2016520 and the rs9794 minor allele of *PPARD* and decreased risk of HTN and additional interactions between these two SNPs. Although the *PPARD* rs7770619 SNP is not equivalent to the SNPs identified in other studies [9], the results of this study indicate that the *PPARD* rs7770619 SNP may represent a critical locus that negatively influences HTN and oxidative stress.

The *PPARD* rs7770619 C>T SNP is considered functional since serum glucose and the *PPARD* rs7770619 C>T polymorphism were significantly associated in both normotensive controls and HTN patients in this study. *PPARD* activation in the liver appears to decrease hepatic glucose output, which contributes to improved glucose control [16]. *PPARD* also appears to have a role in the regulation of fatty acid oxidation in several tissues, including skeletal muscle and adipose tissue [17]. It has been suggested that the mechanisms of action of this gene involve redistribution of the non-esterified fatty acid (NEFA) flux. The increasing oxidative capability draws the NEFA to the muscle to be preferentially oxidized rather than stored in adipose tissue, which leads to a decrease in adipocyte size, enhanced lipolysis, and increased adiponectin secretion [18]. In this study, subjects with the *PPARD* rs7770619 CT genotype showed significantly higher adiponectin concentrations than those with the CC genotype in both the normotensive controls and the HTN patients. Additionally, HTN patients with the CT genotype showed an increase in serum free fatty acid compared with subjects with the CC genotype.

The rs7770619 SNP, showing the features above in this study, is an intron mutation located at chromosome 6:35382265 (GRC38p.12 assembly of the human genome). Introns are involved in regulation of alternative splicing and gene expression [19]; therefore, the rs7770619 C>T polymorphism “may” affect the *PPARD* gene splicing, *PPARD* splice variants generation, and/or PPARD function. So far, however, there is a lack of studies on rs7770619, and studies about an impact of rs7770619 on *PPARD* gene splicing do not exist. Thus, at the present stage of knowledge and information, it is difficult to prove that the intron mutation of rs7770619 “really” influences splicing of *PPARD* gene. Moreover, to the best of our knowledge, no literatures that investigated altered risk of HTN, oxidative stress, or MDA levels according to *PPARD* splice variants exist. Our study only approached the association between *PPARD* rs7770619 C>T polymorphism and the risk of HTN in the Korean cohort; thus, the exact mechanism on development of HTN through the SNP is still unknown. Therefore, attempts to verify the underlying mechanism is needed in the future.

According to the dbSNP (http://www.ncbi.nlm.nih.gov/snp), MAF for rs7770619 SNP is 7.5% in the 1000 Genomes Project (phase 3) and 10.5% in TOPMED. In the present study, MAF of rs7770619 was 2.4% in the whole participants (2.8% and 1.2% for normotensive controls and HTN patients, respectively); somewhat low MAF was shown in the Korean cohort compared to the world’s average. Indeed, MAFs for rs7770619 vary based on ethnic groups. Many African populations have high rs7770619 MAF (> 20%), whereas in Asian populations (especially East Asians such as Korean, Chinese, and Japanese), rs7770619 MAF is generally low; based on the 1000 Genomes Project (phase3) (https://www.ncbi.nlm.nih.gov/variation/tools/1000genomes), in the population of Han Chinese in Beijing (HCB population) and Japanese in Tokyo (JPT population), MAF is 1.5% and 0.5%, respectively. Therefore, considering ethnic origin, MAF for rs7770619 observed in our cohort is not exclusively low. Many studies have reported the ethnic-dependent differences of HTN prevalence; studies have shown that black people are under higher risk of HTN than white people [20–23]. Moreover, Cappuccio et al. [24] showed that the South Asian population had two- to three-fold higher prevalence of HTN than white people, and Holly et al. [23] also showed significant higher odds ratio of HTN in the Chinese population compared with the white population after adjusting confounding factors. In dbSNP, the Caucasian population (CAUC1) shows 8.1% of MAF for rs7770619. Since our results demonstrated that rs7770619 minor T allele is associated with lower risk of HTN, higher risk of HTN in Asian populations whose MAF for rs7770619 is lower than white people makes sense; however, higher prevalence of HTN was also observed in the black population, thus, our results do not entirely correspond with other researches in terms of ethnicity. HTN can be caused by various risks such as genetic, metabolic, and environmental (e.g., dietary habit and physical activity) factors. A complex interaction of these risk factors induces HTN; thus, risk of HTN according to ethnic groups cannot be explained by only one polymorphism. At least, in a Korean population, rs7770619 was found as a novel SNP for HTN risk via a connection with oxidative stress (MDA). In addition, rs7770619 C>T polymorphism has not been studied with regard to oxidative stress and HTN, and this is the first study to identify association between rs7770619 SNP and HTN risk. Therefore, based on the results of our study, gradual expansion of the HTN study in respect of rs7770619 polymorphism is needed.

There are several limitations. First, when interpreting the findings of this study, it should be considered that our results share the limitations of cross-sectional observational studies becuase we evaluated only an association rather than prospective prediction. Second, in terms of conventional genome-wide association study (GWAS), this study did not share the exact analysis method of conventional GWAS, because (1) imputation could not be conducted due to the limit of equipment's calculation capacity and (2) when we selected SNPs, we used FDR correction instead of Bonferroni correction, widely used in conventional GWAS. Bonferroni correction has a very conservative threshold (*p* < 5 × 10^−8^) so that it can cause decrease of statistical power [25, 26]. Recently, indeed, not only Bonferroni correction but also FDR correction has been used in common practice [27, 28]. Third, unexpectedly, low MAF generated a too small sample size of individuals with rs7770619 CT genotype; thus, in Fig. 1, a subset analysis with the HTN without treatment group (for control of HTN medication effects) according to the rs7770619 genotypes could not be performed due to a problem of statistical power. Lastly, we specifically focused on a representative group of Korean subjects. Therefore, our results cannot be generalized to other ethnicities, age groups, or geographical groups. Taken together, further study needs to be conducted by exact manner of GWAS with a cohort having a much larger sample size to confirm replication of the results observed in this study. Despite these limitations, our results show interesting associations between the *PPARD* rs7770619 CT genotype, a decreased risk of HTN, and decreased oxidative stress. These results suggest that the *PPARD* rs7770619 C>T SNP is a novel candidate gene for HTN through the association between *PPARD* and MDA, a biomarker of oxidative stress.

## Conclusion

According to the individuals’ *PPARD* rs7770619 genotype, the risk of HTN development is predictable via association between *PPARD* rs7770619 SNPs and MDA. Reducing MDA in subjects with *PPARD* rs7770619 CC genotype is necessary to decrease the risk of HTN development. Therefore, by analyzing personal genetic background, MDA, the oxidative stress marker, can be considered as a therapeutic target of HTN in a Korean population.

## Additional file

Additional file 1: **Table S1.** Top 10 SNPs associated with plasma MDA. **Figure S1.** Flow of the study participants. (DOCX 112 kb)

## Abbreviations

BP: Blood pressure
CI: Confidence interval
FDR: False discovery rate
GWAS: Genome-wide association study
HDL: High-density lipoprotein
HOMA: Homeostatic model assessment
hs-CRP: High-sensitivity C-reactive protein
HTN: Hypertension
HWE: Hardy-Weinberg equilibrium
IR: Insulin resistance
K-CHIP: Korean Chip
LDL: Low-density lipoprotein
MDA: Malondialdehyde
NEFA: Non-esterified fatty acid
OR: Odds ratio
PPAR: Peroxisome proliferator-activated receptor
PPARD: Peroxisome proliferator-activated receptor-δ
ROS: Reactive oxygen species
SNP: Single-nucleotide polymorphism
TC: Total cholesterol
TG: Triglyceride
