# Supplementary material for: Associations between hypertension and the peroxisome proliferator-activated receptor-δ (PPARD) gene rs7770619 C>T polymorphism in a Korean population
Source: Hum Genomics. 2018 Jun 18;12:28. doi: 10.1186/s40246-018-0162-6 (PMC6006737; doi:10.1186/s40246-018-0162-6)
Supplement: Supplementary file 1 — Table S1. Top 10 SNPs associated with plasma MDA. Figure S1. Flow of the study participants. (DOCX 112 kb) [file 40246_2018_162_MOESM1_ESM.docx]

**Associations between hypertension and the peroxisome proliferator-activated receptor-δ (*PPARD*) gene rs7770619 C>T polymorphism in a Korean population**

**Supplementary materials**

**Table S1. Top 10 SNPs associated with plasma MDA**

| **No.** | **CHR** | **Associated gene** | **SNP (rs number)** | ***p*** | ***q*** |
| --- | --- | --- | --- | --- | --- |
| 1 | 18 | *GNAL* | - | 2.92E-09 | 0.001 |
| 2 | 2 | *FAM124B, CUL3* | rs74272770 | 6.63E-09 | 0.001 |
| 3 | 22 | *TBX1* | - | 5.62E-08 | 0.005 |
| 4 | 9 | *SHB* | - | 5.99E-08 | 0.005 |
| 5 | 19 | *APLP1* | - | 8.49E-08 | 0.005 |
| 6 | 20 | *ADRA1D* | rs61759840 | 9.86E-08 | 0.005 |
| 7 | 11 | *LOC101929011, BUD13, LINC00900* | rs12361543 | 1.07E-07 | 0.005 |
| 8 | 9 | *EPB41L4B* | - | 1.23E-07 | 0.005 |
| 9 | 15 | *SMAD6* | - | 1.41E-07 | 0.005 |
| 10 | 6 | ***PPARD*** | **rs7770619** | 1.43E-07 | 0.005 |

According to dbSNP (http://www.ncbi.nlm.nih.gov/snp), only 4 SNPs have the rs number. *p*-values derived from a linear regression analysis. *q*-values were adjusted *p*-value that controls the false discovery rate (FDR); and *q*<0.05 was considered to be statistically significant.


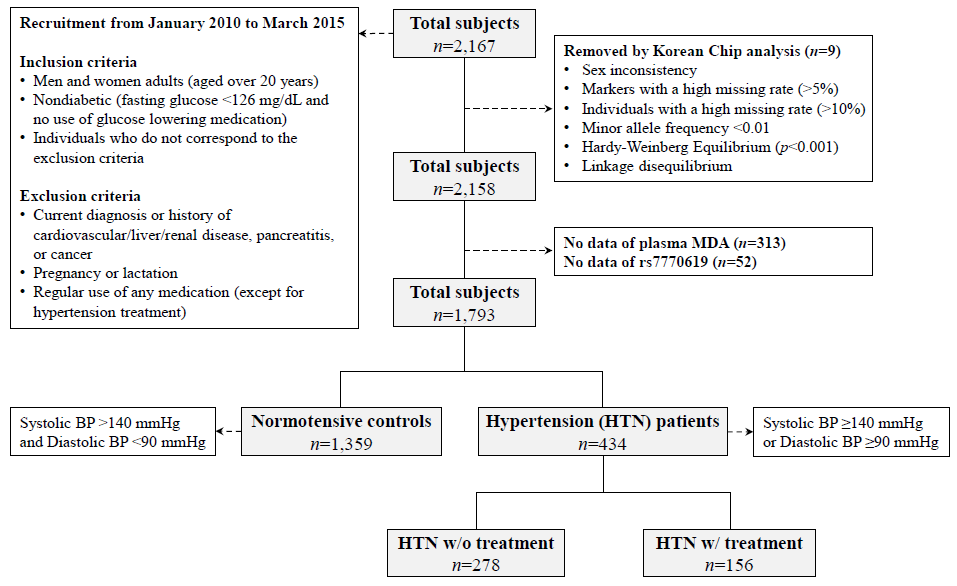


**Figure S1. Flow of the study participants**
